# Methodological factors affecting gas and methane production during in vitro rumen fermentation evaluated by meta-analysis approach

**DOI:** 10.1186/s40104-016-0094-8

**Published:** 2016-06-14

**Authors:** Laura Maccarana, Mirko Cattani, Franco Tagliapietra, Stefano Schiavon, Lucia Bailoni, Roberto Mantovani

**Affiliations:** Department of Comparative Biomedicine and Food Science (BCA), University of Padova, Viale dell’Università 16, 35020 Legnaro (PD), Italy; Department of Agronomy, Food, Natural resources, Animals and Environment (DAFNAE), University of Padova, Viale dell’Università 16, 35020 Legnaro (PD), Italy

**Keywords:** Experimental factors, Gas production, In vitro rumen fermentation, Meta-analysis, Methane production

## Abstract

Effects of some methodological factors on in vitro measures of gas production (GP, mL/g DM), CH_4_ production (mL/g DM) and proportion (% CH_4_ on total GP) were investigated by meta-analysis. These factors were considered: pressure in the GP equipment (0 = constant; 1 = increasing), incubation time (0 = 24; 1 = ≥ 48 h), time of rumen fluid collection (0 = before feeding; 1 = after feeding of donor animals), donor species of rumen fluid (0 = sheep; 1 = bovine), presence of N in the buffer solution (0 = presence; 1 = absence), and ratio between amount of buffered rumen fluid and feed sample (BRF/FS; 0 = ≤ 130 mL/g DM; 1 = 130–140 mL/g DM; 2 = ≥ 140 mL/g DM). The NDF content of feed sample incubated (NDF) was considered as a continuous variable. From an initial database of 105 papers, 58 were discarded because one of the above-mentioned factors was not stated. After discarding 17 papers, the final dataset comprised 30 papers (339 observations). A preliminary mixed model analysis was carried out on experimental data considering the study as random factor. Variables adjusted for study effect were analyzed using a backward stepwise analysis including the above-mentioned variables. The analysis showed that the extension of incubation time and reduction of NDF increased GP and CH_4_ values. Values of GP and CH_4_ also increased when rumen fluid was collected after feeding compared to before feeding (+26.4 and +9.0 mL/g DM, for GP and CH_4_), from bovine compared to sheep (+32.8 and +5.2 mL/g DM, for GP and CH_4_), and when the buffer solution did not contain N (+24.7 and +6.7 mL/g DM for GP and CH_4_). The increase of BRF/FS ratio enhanced GP and CH_4_ production (+7.7 and +3.3 mL/g DM per each class of increase, respectively). In vitro techniques for measuring GP and CH_4_ production are mostly used as screening methods, thus a full standardization of such techniques is not feasible. However, a greater harmonization of analytical procedures (i.e., a reduction in the number of available protocols) would be useful to facilitate comparison between results of different experiments.

**Electronic supplementary material:**

The online version of this article (doi:10.1186/s40104-016-0094-8) contains supplementary material, which is available to authorized users.

## Introduction

In recent years in vitro gas production (GP) technique has been applied routinely to evaluate the nutritional value of ruminant feeds. The most diffused techniques were designed to measure GP from feed samples incubated in glass syringes at atmospheric pressure [1] or in fermentation vessels where gas is measured over the whole incubation time [2] or regularly vented at fixed times [3] or at fixed pressure [4, 5]. More recently, such equipment has been adapted to determine the composition of gases, particularly methane (CH_4_) produced from in vitro rumen fermentation [6–8]. The current literature [9, 10] encompasses reviews which explored the effect of various factors influencing in vitro GP values, as procedures used to collect and to treat rumen fluid [11], the composition of the buffer [12], the type of GP equipment [13–15] and the ratios between fermentation fluid and feed sample size [8]. On the contrary, to our knowledge, no literature reviews have been produced to investigate factors affecting CH_4_ measures obtained in vitro.

The objective of the present study was to evaluate factors affecting in vitro GP and CH_4_ production by means of the meta-analysis approach.

## Materials and methods

### Literature search

An as wide as possible literature search was conducted using search generators of public data (i.e. Web of knowledge, Google scholar, Science direct, and Scopus) and contacts with researchers working in this field, to find scientific papers reporting data of gas and CH_4_ production obtained from in vitro fermentation of feeds or diets commonly used for ruminants. The web searches were conducted using the following keywords in different combinations: in vitro technique, rumen fermentation, gas production, methane production and ruminants.

### Factors selected

The search strategy aimed at selecting articles focusing on the study of specific factors known to exert notable effects on in vitro gas and CH_4_ production. Specifically, the following factors were considered: the pressure in the GP equipment used, the incubation time, the collection time of rumen fluid, the donor species, the presence or absence of N in the buffer solution added to the rumen fluid, the NDF content of incubated feed samples, the amount of buffer solution (B), the amount of rumen fluid (RF), and the amount of feed sample incubated (FS). The correction of in vitro data for “blank” samples, despite its relevance, was not considered as most papers did not provide this information. The chemical composition of diet fed to animals used as donors of rumen fluid was not taken into account because of huge variability among studies. In any case, the review of [10] indicated that the diet of donor animals does not have a significant effect on in vitro GP, provided that it is able to enhance a sufficient microbial activity to sustain GP. This prerequisite is commonly ensured in all studies. Agitation (or not) of bottles during the incubation was not taken into account because this factor has been reported as having minor effects on in vitro GP [10]. Moreover, this information is absent in many of the considered manuscripts.

### Inclusion and exclusion of literature for the study and building of the starting dataset

A total of 105 scientific papers were identified and initially screened for acceptability by checking if all publications reported the above-mentioned information. To be included in the database, papers had to report all values of GP, CH_4_, and CH_4_ proportion on total GP (% CH_4_ on total GP), or at least two of them, so that the third variable could be calculated. Results of studies carried out by using continuous (i.e. dual flow) or semi-continuous (i.e. RUSITEC^®^) GP equipment were not considered in the study. Experiments conducted using alternative inocula (i.e. faeces) instead of rumen fluid were excluded from the database, as well as studies that did not declare the time of rumen fluid collection. After discarding 58 out of the 105 papers (Table 1), a starting dataset was built considering 47 articles published over the last 12 yr, accounting for a total of 393 observations (Table 2). Such observations included only control treatments, defined as feeds or diets incubated alone. Observations referred to effects of additives on in vitro GP and CH_4_ production were not considered, to avoid possible further confounding effects due to the presence of such compounds. In the dataset some unpublished data of completed studies were included [15].

**Table 1:** List of references excluded from the meta-analysis. Additional file 1

References	Reason for exclusion
Salem, 2012	Only gas production was measured
Abarghuei et al., 2014	
Rodrigues et al., 2014	
Salem et al., 2014	
Elghandour et al., 2015	
Lavrencic et al., 2015	
Rojas Hernandez et al., 2015	
Rossi et al., 2001	Only methane (CH_4_) production data were reported and not the total gas production (GP) or CH_4_ proportion on total GP (% CH_4_ on total GP)
Wallace et al., 2006	
Wood et al., 2009	
Becker and van Wikselaar, 2011	
Cao et al., 2012	
Castro-Montoya et al., 2012	
Poulsen et al., 2012	
O’Brien et al., 2013	
Rira et al., 2015	
Aemiro et al., 2016	
Lovett et al., 2004	At least one investigating factor was missing
Hu et al., 2005	
Tavendale et al., 2005	
Lovett et al., 2006	
Patra et al., 2006	
Hassim et al., 2010	
Kamalak et al., 2011	
Sun et al., 2011	
Baraka and Abdl-Rahman, 2012	
Blanco et al., 2012	
Banik et al., 2013	
Kim et al., 2013	
Lin et al., 2013	
Naumann et al., 2013	
Durmic et al., 2014	
Nanon et al., 2014	
Castagnino et al., 2015	
Cobellis et al., 2015	
Copani et al., 2015	
Jayanegara et al., 2015	
Liu et al., 2015	
Pirondini et al., 2015	
Qiao et al., 2015	
Rajkumar et al., 2015	
Saminathan et al., 2015	
Theart et al., 2015	
Serment et al., 2016	
Anele et al., 2011	Methane production data were indirectly predicted
Zhang et al., 2011	
Meale et al., 2012	Methane production data were indirectly predicted
Pang et al., 2014	
Polyorach et al., 2014	
Gemeda and Hassen, 2015	
Ungerfeld et al., 2007	Control data of feed sample treatment were missing
Hart et al., 2008	
Wang et al., 1998	GP and methane data were obtained using continuous or semi-continuous apparatus
Amelchanka et al., 2010	
Soliva et al., 2011	
Williams et al., 2011	
Li et al., 2013	
Wischer et al., 2013

**Table 2:** List of preliminary references considered with their respective description of factors selected as possible sources of variation on total gas production (GP), methane (CH_4_) production and proportion (% CH_4_ on total GP) (*n* = 393 observations, 47 papers). Additional file 2

References	N^a^	Pressure^b^	Incubation time^c^, h	Donor species^d^	Collection time^e^	N buffer^f^	NDF, g/kg^g^	B, mL^h^	RF, mL^i^	FS, g DM^j^
Lila et al., 2003	6	increasing	6; 24	bovine	before feeding	yes	0-473	20	10	0.18
Lila et al., 2004	1	increasing	6	bovine	before feeding	yes	466	20	10	0.18
Getachew et al., 2005	28	constant	6; 24; 48; 72	bovine	after feeding	yes	250-315	20	10	0.18
Longo et al., 2006	8	constant	24	sheep	before feeding	yes	240-769	50; 80	20; 25	0.46-0.92
Bodas et al., 2008	11	increasing	24	sheep	before feeding	yes	450	40	10	0.55
Garcia-Gonzales et al., 2008a	2	increasing	24	sheep	before feeding	yes	440	40	10	0.45
Garcia-Gonzales et al., 2008b	1	increasing	24	sheep	before feeding	yes	386	40	10	0.52
Macheboeuf et al., 2008	8	increasing	16	sheep	before feeding	no	262	25	15	0.37
Soliva et al., 2008	26	constant	24	bovine	before feeding	yes	254-583	10	20	0.28
Holtshausen et al., 2009	1	increasing	24	bovine	after feeding	no	347	15	5	0.50
Martínez et al., 2010	8	constant	8; 24	sheep	before feeding	yes	374-499	32	8	0.37
Sallam et al., 2010	3	increasing	24	sheep	before feeding	no	547-616	50	25	0.46
Xu et al., 2010	15	increasing	24	bovine	after feeding	yes	126-749	42	8	0.55
Araujo et al., 2011	1	increasing	16	sheep	before feeding	yes	203	50	25	0.46
Avila et al., 2011	1	increasing	48	bovine	after feeding	no	385	18	6	0.50
Guglielmelli et al., 2011	5	increasing	48	bovine	slaughterhouse	no	391-523	74	5	0.93
Lee et al., 2011	2	increasing	24	bovine	before feeding	yes	116-451	40	10	0.43-0.45
Navarro-Villa et al., 2011a	27	increasing	24	bovine	before feeding	yes	187-871	33-43	7-16	0.28-0.64
Navarro-Villa et al., 2011b	4	increasing	24	bovine	before feeding	no	396-498	40	10	0.46
Pellikaan et al., 2011	11	increasing	72	bovine	after feeding	yes	25-648	40	20	0.45-0.48
Purcell et al., 2011a	9	increasing	24	bovine	before feeding	no	351-426	40	10	0.46
Purcell et al., 2011b	7	increasing	24	bovine	before feeding	no	458-643	40	10	0.46
Theodoridou et al., 2011	4	increasing	24	sheep	before feeding	no	253-526	26.6	13.3	0.55
Zhang and Yang, 2011	1	constant	48	bovine	after feeding	yes	524	50	25	0.46
Amaro et al., 2012	1	increasing	24	bovine	slaughterhouse	yes	383	33	17	0.39
Carrasco et al., 2012	1	increasing	17	bovine	slaughterhouse	no	179	32	8	0.40
Garcia-Gonzales et al., 2012	1	increasing	12	sheep	after feeding	yes	0	40	10	0.46
Hassanat et al., 2012	1	increasing	24	bovine	after feeding	no	331	17	3	0.18
Pirondini et al., 2012	2	increasing	24	bovine	before feeding	yes	321-492	20	10	0.23
Ramin and Huhtanen, 2012	4	constant	48	bovine	after feeding	no	570	48	12	0.29-1.15
Boguhn et al., 2013	8	constant	24	sheep; bovine	before feeding	no	375-398	20	10	0.11
Geerkens et al., 2013	3	constant	24	bovine	before feeding	no	169-520	20	10	0.11
Hansen et al., 2013	1	constant	48	bovine	before feeding	yes	465	60	30	0.46
Narvaez et al., 2013	3	increasing	48	bovine	after feeding	no	372	27	13	0.46
Patra and Yu, 2013a	1	increasing	24	bovine	after feeding	yes	292	30	10	0.37
Patra and Yu., 2013b	2	increasing	24	bovine	after feeding	yes	290-416	30	10	0.37
Ramin et al., 2013	32	constant	24; 48	bovine	after feeding	yes	249-613	40	20	0.46
Tuyen et al., 2013	4	increasing	48	bovine	after feeding	yes	714-929	40	20	0.42-0.52
Bezabih et al., 2014	58	increasing	24; 72	bovine	after feeding	yes	184-684	40	20	0.46
Cattani et al., 2014	20	increasing constant	24	bovine	before feeding	yes	106-591	40	20	0.36-0.38
Elghandour et al., 2014	4	increasing	72	bovine	before feeding	yes	459-557	40	10	0.92
Kim et al., 2014	2	increasing	24	bovine	after feeding	yes	137-519	80	20	0.28
O’Brien et al., 2014	22	increasing	24	bovine	before feeding	yes	326-426	40	10	0.46
Pal et al., 2014	8	constant	24	sheep	before feeding	yes	401-518	20	10	0.18
Hatew et al., 2015	4	constant	24	bovine	before feeding	yes	378-441	40	20	0.46
Pal et al., 2015	18	constant	24	sheep	before feeding	yes	266-523	20	10	0.18
Ramin et al., 2015	3	constant	48	bovine	after feeding	yes	239-570	40	20	0.93

^a^ *N* = number of observations per article

^b^Pressure = pressure produced in the GP equipment used

^c^Incubation time = duration of incubation

^d^Donor species = donor species of rumen fluid

^e^Collection time = origin of rumen fluid: if it was collected before (before feeding or at slaughterhouse) or after feeding of donor animals

^f^N buffer = presence of N in the buffer solution

^g^NDF, g/kg = actual NDF content of feed samples used

^h^B, mL = buffer incubated

^i^RF, mL = rumen fluid incubated

^j^FS, g DM = feed sample incubated

### Data harmonization

Because of the heterogeneity in GP and CH_4_ values reported among publications, data were adjusted to a uniform scale. All GP data were transformed to mL per gram of incubated DM. Likewise CH_4_ values were converted and expressed in terms of total CH_4_ production (mL per gram of incubated DM) and as a proportion of total GP (mL per 100 mL of total GP). When not otherwise specified, the weight of the sample was considered as fed (on a wet basis). To reconcile the weight of a feed sample into g of incubated DM, values were corrected using the DM content of each sample. When DM was not indicated, a value of DM equal to 920 g/kg was used, corresponding to the general DM mean content of feed samples included in the dataset. When papers presented GP and CH_4_ values in terms of moles, a correction was adopted using Gay-Lussac’s law, assuming that 1 mol was equivalent to 25.6 L of gas under atmospheric pressure and temperature conditions of GP equipment (39 °C). In the case of [16], 1 mol of gas was considered to be equivalent to 25.4 L, as indicated by the authors. To convert values of GP and CH_4_ expressed as mL per gram of OM, the DM and ash contents of feed samples were considered. When CH_4_ values were expressed in mg/g, these values were converted into mL using Gay-Lussac’s law and considering the molecular weight of CH_4_. When CH_4_ values were expressed as mmol/L, they were reconciled considering Gay-Lussac’s law and GP values; when CH_4_ data were expressed as mL/L they were reconciled considering only values of GP.

### Evaluation of the preliminary dataset (47 papers; 393 observations)

Variables such as the pressure in the GP equipment used (constant vs. increasing; 162 vs. 231 observations, respectively), the incubation time (≤24 vs. ≥ 48 h; 297 vs. 96 observations, respectively), the collection time of rumen fluid (before or after feeding of donor animals; 224 vs. 169 observations, respectively), the donor species (sheep vs. bovine; 77 vs. 316 observations, respectively), and the presence of N in the buffer solution (presence vs. absence; 331 vs. 62 observations, respectively) were all coded as dichotomous variables, i.e., 0 or 1 in respective order. Syringes and vented bottles were considered as equipment working at constant pressure, whereas closed bottles were considered as apparatus operating at increasing pressure. Data of rumen fluid obtained at slaughterhouse were considered as collected before feeding and, thus, coded as 0. The actual NDF content of feed sample (426 ± 168.5 g/kg), the amount of buffer (35.3 ± 11.79 mL), the amount of rumen fluid (13.9 ± 5.34 mL), and the amount of feed sample incubated (0.41 ± 0.170 g DM) were initially treated as possible continuous variables. The NDF content of feeds was the only chemical constituent considered because: i) it was the only analytical measure reported by all scientific papers taken into account; ii) the NDF fraction is commonly considered a good descriptor of fermentation properties of feeds and/or diets [17] and it is strictly related with gas and CH_4_ production [18].

Two preliminary analyses of data were carried out. The first aimed at investigating the best classification system for the incubation time variable. Two classes of incubation time (24 or ≥ 48 h) were chosen as the final outcome of the first preliminary investigation. The second analysis was carried out to test the possibility of treating as continuous variables the amounts of buffer, rumen fluid, and feed sample used for in vitro tests. Because of their low variability within experiment, the three variables were not run separately in the statistical model, but they were included as the ratio between the buffered rumen fluid (mixture of buffer solution and rumen fluid) and the feed sample, here defined as the BRF/FS ratio. This choice was further motivated by the fact that this ratio has a relevant effect on in vitro GP and GP kinetics [10, 19]. In the present study the BRF/FS ratio was coded into three classes (<130 vs. 130–140 vs. > 140 mL/g DM; 173, 105, and 115 observations, respectively) as 0 or 1 or 2, in respective order.

### Data cleansing to obtain the final dataset

The final dataset submitted to the statistical analysis accounted for only 339 (corresponding to 30 scientific papers) out of the 393 initial observations. Firstly, 26 observations were excluded as they were obtained using an incubation time shorter than 24 h. After that, according to the indications suggested by [20] for meta-analysis, other 28 observations were discarded as: i) the continuous variable considered (NDF) was constant in the study (20 observations); and ii) studies accounted for a single observation (8 observations).

### Statistical analysis

The latter dataset was analyzed using a mixed model analysis accounting for the random study effect, with the scope of eliminating possible confounding effects due to differences within and across studies, as suggested by [20].

To overcome a possible over-parameterization of the mixed model, a first analysis accounting for the random study effect via the PROC MIXED of SAS [21] was carried out, considering a variance component (TYPE = VC) covariance structure [20]. To take into account the different accuracies among studies as well, all dependent variables were weighed by the inverse of the squared standard error divided by the mean of all the squared standard errors, as suggested by [20]. At a later stage, data of GP and CH_4_, adjusted for the heterogeneity due to different studies, i.e., study effect [20], were analyzed using the backward elimination technique [22] of SAS (PROC REG; [21]). The exit level for each variable, i.e., the threshold of significance for excluding a variable from the model, was set at *P* > 0.10. Multi-collinearity among predictor variables was analyzed through the variance inflation factor (VIF). According to [23], the multi-collinearity can be considered not significantly inflated when the VIF is lower than 10. The collinearity among explanatory variables included in the multivariate stepwise analysis, was calculated in terms of minimum condition index, to exclude the presence of dependencies (i.e., common variance explained) among the considered variables. According to [24], there are no dependencies when the minimum condition index is lower than 30.

### Description of the preliminary dataset (47 papers; 393 observations)

The list of references excluded from the meta-analysis and the reasons for exclusion are given in Table 1. The references entering the preliminary dataset and the corresponding description of factors are listed in Table 2. In most of the experiments (32 out of a total of 47), fermentations occurred in conditions of increasing pressure, and gas was accumulated into the GP system during the incubation; 14 studies were conducted at constant pressure, by a regular venting of fermentation gases, whereas one study applied both constant and increasing pressure. The majority of the in vitro experiments (27) were stopped at 24 h; in 5 studies, fermentations lasted less than 24 h, whereas 10 studies used an incubation time ≥ 48 h. In 5 researches, different incubation times were compared. Rumen fluid used for in vitro tests was preferentially collected from bovine (34 studies), whereas 12 experiments used sheep as donors. One study compared rumen fluid collected from sheep or bovine. In most of the cases rumen fluid was collected before feeding of donor animals (before feeding in 27 studies; at slaughterhouse in 3 studies); however, in a relevant number of cases (i.e., 17 studies) rumen fluid was collected after feeding of donors. In a large number of experiments (32 out of a total of 47), rumen fluid was mixed with a buffer solution containing N. The NDF content of feed samples incubated showed a high variability, ranging from a very low (0 g/kg, for potato starch and corn starch) to an extremely high value (929 g/kg, for sugarcane bagasse). With the exception of two studies Additional file 1: (Longo et al., 2006; Navarro-Villa et al., 2011a), the amounts of buffer and rumen fluid used in the study presented no variability, whereas in 7 papers different amounts of feed sample were tested.

## Results

### Description of the final dataset (30 papers; 339 observations)

The mean and standard deviation (s.d.) values of in vitro GP, CH_4_ production and proportion, obtained considering the final dataset, are given in Table 3.

**Table 3:** Means and standard deviation (s.d.) of total gas production (GP), methane (CH_4_) production and proportion (% CH_4_ on total GP) of the 339 observations belonging to 30 reference used for the final analysis. Additional file 2

References	No. ^a^	GP, mL/g DM		CH_4_, mL/g DM		CH_4_, % on total GP	
		mean	s.d.	mean	s.d.	mean	s.d.
Lila et al., 2003	3	200	31.0	76.0	23.26	37.6	6.05
Getachew et al., 2005	21	235	20.9	53.8	15.65	22.6	5.20
Longo et al., 2006	8	131	70.5	24.8	17.33	18.0	4.14
Soliva et al., 2008	26	129	49.5	15.0	10.46	10.6	3.72
Martínez et al., 2010	4	480	17.7	45.2	5.41	9.4	0.78
Sallam et al., 2010	3	72	33.8	7.3	3.61	10.1	0.61
Xu et al., 2010	15	163	75.5	16.9	3.90	12.1	4.51
Guglielmelli et al., 2011	5	141	16.6	24.5	5.23	17.3	2.32
Lee et al., 2011	2	194	65.8	23.8	8.27	12.3	0.07
Navarro-Villa et al., 2011a	27	141	59.9	20.5	8.78	14.8	2.58
Navarro-Villa et al., 2011b	4	158	14.5	35.3	1.80	22.4	0.95
Pellikaan et al., 2011	11	276	70.7	47.3	9.65	17.5	2.22
Purcell et al., 2011a	9	183	7.7	25.1	1.04	13.7	0.36
Purcell et al., 2011b	7	171	17.5	31.3	3.45	18.3	0.48
Theodoridou et al., 2011	4	133	5.3	33.0	6.60	24.5	4.49
Pirondini et al., 2012	2	243	40.3	40.3	5.73	16.6	0.35
Boguhn et al., 2013	8	292	17.9	44.2	5.53	15.1	1.42
Geerkens et al., 2013	3	307	52.4	50.7	9.29	16.5	0.41
Patra and Yu., 2013b	2	191	12.5	77.5	4.60	40.6	5.09
Ramin et al., 2013	32	223	77.5	36.2	9.83	16.9	3.03
Tuyen et al., 2013	4	95	41.8	17.6	7.17	19.1	2.39
Bezabih et al., 2014	58	200	39.5	41.9	11.91	20.9	4.19
Cattani et al., 2014	20	192	77.6	23.0	8.03	12.3	1.28
Elghandour et al., 2014	4	224	51.8	17.0	7.62	7.4	2.91
Kim et al., 2014	2	337	120.4	22.0	9.22	6.4	0.43
O’Brien et al., 2014	22	201	2.8	34.1	4.56	17.0	2.19
Pal et al., 2014	8	147	22.5	35.1	6.55	24.5	6.37
Hatew et al., 2015	4	312	23.8	54.5	5.78	17.5	0.63
Pal et al., 2015	18	101	33.3	11.5	2.57	12.2	3.11
Ramin et al., 2015	3	275	43.7	36.5	5.97	13.3	0.46

^a^No. = number of observations per article

Table 4 shows the mean and s.d. values of in vitro GP (mL/g DM), CH_4_ production (mL/g DM) and CH_4_ proportion on total GP for the different possible sources of variation taken into account for the 30 literature papers considered in the meta-analysis. The use of GP systems working at constant pressure (with gas venting), incubation time ≥ 48 h, rumen fluid collected from bovine after feeding, and a BRF/FS ratio included between 130 and 140 mL/g DM, determined an increase of GP values. Measures of CH_4_ were higher with incubation time ≥ 48 h, with rumen fluid collected after feeding of donor animals, and with a BRF/FS ratio  > 140 mL/g DM. When CH_4_ data were expressed as proportion on total GP, values resulted greater at increasing pressure (+12.1 % compared to constant pressure) and at increasing incubation times (+29.5 % with time ≥ 48 h compared to 24 h), when collection of rumen fluid was performed after feeding (+25.8 % compared to before feeding), and when BRF/FS was > 140 mL/g DM (+30.6 % and +12.9 %, compared to BRF/FS < 130 and 130 ≤ BRF/FS ≤ 140 mL/g DM, respectively).

**Table 4:** Descriptive statistics of total gas production (GP), methane (CH_4_) production and proportion (% CH_4_ on total GP) for the main sources of variation analyzed in the multivariate stepwise analysis after correction for the study effect (*n* = 339 observations, 30 papers)

Main factors	No^a^	GP, mL/g DM		CH_4_, mL/g DM		CH_4_, % on total GP	
		mean	s.d.^b^	mean	s.d.	Mean	s.d.
Pressure							
Constant	145	198	94.4	31.8	17.94	15.7	5.76
Increasing	194	185	61.4	32.4	15.29	17.6	5.95
Incubation time, h							
24	253	178	77.9	27.6	14.20	15.6	5.59
≥ 48	86	227	63.1	45.3	15.63	20.2	5.60
Collection time							
Before feeding	191	174	81.5	26.3	14.58	15.1	5.47
After feeding	148	212	65.9	39.7	15.68	19.0	5.79
Donor species							
Sheep	49	162	116.6	24.5	15.60	15.9	6.40
Bovine	290	195	67.7	33.4	16.29	16.9	5.85
N in the buffer							
Presence	296	191	78.4	32.2	17.07	16.8	6.18
Absence	43	190	71.0	31.5	11.52	16.7	3.82
BRF/FS^c^							
< 130 mL/g DM	134	172	77.6	25.0	12.74	14.7	5.54
130-140 mL/g DM	105	217	65.4	40.8	12.00	19.2	4.08
> 140 mL/g DM	100	187	81.5	32.5	20.17	17.0	7.02

^a^No = number of observations accounted in each class

^b^s.d. = standard deviation of means

^c^BRF/FS = ratio between buffered rumen fluid and feed sample

Table 5 shows the predictive equations for in vitro GP (mL/g DM), CH_4_ production (mL/g DM) and CH_4_ proportion (% on total GP). The predictive equations were the following:

**Table 5:** Outcome of the backward stepwise multivariate regression analysis on predicted values obtained by correcting for the study effect and adjusting raw data for different accuracies^a^ of the total gas production (GP), methane (CH_4_) production and proportion (% CH_4_ on total GP)

Items	GP, mL/g DM		CH_4_, mL/g DM		CH_4_, % on total GP	
	estimate ± SE	*P*	estimate ± SE	*P*	estimate ± SE	*P*
Intercept	141.0 ± 5.24	<0.01	21.8 ± 2.22	<0.01	15.0 ± 0.31	<0.01
Pressure^b^	-	-	-	-	0.9 ± 0.17	<0.01
Incubation time^c^	7.9 ± 3.30	0.018	4.2 ± 1.40	<0.01	-	-
Collection time^d^	26.4 ± 3.21	<0.01	9.0 ± 1.37	<0.01	1.2 ± 0.19	<0.01
Donor species^e^	32.9 ± 3.95	<0.01	5.3 ± 1.68	<0.01	-	-
N in the buffer^f^	24.7 ± 3.86	<0.01	6.7 ± 1.64	<0.01	0.7 ± 0.23	<0.01
BRF/FS^g^	7.7 ± 1.59	<0.01	3.3 ± 0.67	<0.01	0.3 ± 0.10	<0.01
NDF, g/kg DM^h^	−0.02 ± 0.007	<0.01	−0.009 ± 0.0031	<0.01	-	-
R^b^	0.48		0.34		0.27	
Max VIF^i^	1.76		1.76		1.77	
Max condition index^j^	11.11		11.11		11.88	

^a^Adjustment for different accuracies of measurements in different studies was carried out by weighing raw data by the inverse of the squared standard error divided by the mean of all the squared standard errors (St-Pierre, 2001 [20])

^b^class 0 = constant or class 1 = increasing pressure

^c^class 0 = 24 h; class 1 = ≥ 48 h of incubation

^d^class 0 = before feeding of donor animals or at slaughterhouse; class 1 = after feeding of donor animals

^e^species used as donor of rumen fluid; class 0 = sheep; class 1 = bovine

^f^class 0 = presence; class 1 = absence of N in the buffer

^g^BRF/FS = (buffered rumen fluid and feed sample ratio) class 0 = <130 mL/g DM; class 1 = 130–140 mL/g DM; class 2= >140 mL/g DM)

^h^actual NDF content of feed sample used: treated as continuous variable

^i^VIF = variance inflation index. When value is less than 10, the predictor variables show no significant multicollinearity

^j^collinearity index. When value is less than 30, the variables tested are independent

$$ \mathrm{G}\mathrm{P}\ \left(\mathrm{mL}/\mathrm{g}\ \mathrm{D}\mathrm{M}\right) = 141.0 + 7.9 \times \mathrm{IT} + 26.4 \times \mathrm{C}\mathrm{T} + 32.8 \times \mathrm{D}\mathrm{S} + 24.7 \times \mathrm{N} + 7.7 \times \mathrm{B}\mathrm{R}\mathrm{F}/\mathrm{F}\mathrm{S}\ \hbox{--}\ 0.02 \times \mathrm{N}\mathrm{D}\mathrm{F} $$

$$ {\mathrm{CH}}_4\left(\mathrm{mL}/\mathrm{g}\ \mathrm{D}\mathrm{M}\right) = 21.8 + 4.2 \times \mathrm{IT} + 9.0 \times \mathrm{C}\mathrm{T} + 5.2 \times \mathrm{D}\mathrm{S} + 6.7 \times \mathrm{N} + 3.3 \times \mathrm{B}\mathrm{R}\mathrm{F}/\mathrm{F}\mathrm{S}\ \hbox{--}\ 0.009 \times \mathrm{N}\mathrm{D}\mathrm{F} $$

$$ {\mathrm{CH}}_4\left(\%\ \mathrm{on}\ \mathrm{total}\ \mathrm{G}\mathrm{P}\right) = 15.0 + 0.9 \times \mathrm{P}\mathrm{R} + 1.2 \times \mathrm{C}\mathrm{T} + 0.7 \times \mathrm{N} + 0.3 \times \mathrm{B}\mathrm{R}\mathrm{F}/\mathrm{F}\mathrm{S} $$

where PR = pressure conditions in the GP system (0 = constant; 1 = increasing); IT = incubation time (0 = 24 h; 1 = ≥ 48 h); CT = collection time of rumen fluid (0 = before feeding; 1 = after feeding of donors); DS = donor species of rumen fluid (0 = sheep; 1 = bovine); N = nitrogen in the buffer (0 = presence; 1 = absence); BRF/FS = buffered rumen fluid and feed sample ratio (0 = ≤ 130 mL/g DM; 1 = 130–140 mL/g DM; 2 = ≥ 140 mL/g DM); and NDF = NDF content of feed sample incubated (g/kg DM).

The values of GP and CH_4_ were influenced by IT (*P* = 0.018 and *P* = 0.003, for GP and CH_4_, respectively), CT (*P* < 0.001 for both, in the same order), DS (*P* < 0.001 and *P* = 0.003, in the same order), N (*P* < 0.001 for both), BRF/FS (*P* < 0.001 for both), and NDF (*P* = 0.005 for both) (Table 5). Values of CH_4_ proportion were influenced by PR (*P* < 0.001), CT (*P* < 0.001), N (*P* = 0.005), and BRF/FS (*P* = 0.002). Values of R^2^ were 0.48, 0.34, and 0.27 for GP, CH_4_ production and proportion, respectively.

For all analyzed factors, the maximum VIF was lower than 10 (Table 5). The collinearity among explanatory variables, expressed as a maximum condition index was lower than 30, ranging from 11.11 to 11.88 (Table 5).

**Fig. 1:**
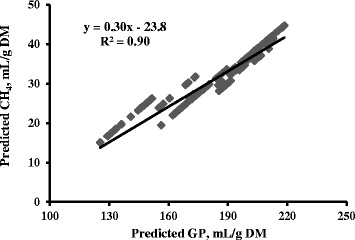
Relationship between gas (GP) and methane (CH_4_) productions using the predicted values obtained from the mixed model analysis aimed to removing the study effect (i.e., the heterogeneity of variance among studies) and considering also the correction of raw data for the different accuracies

**Fig. 2:**
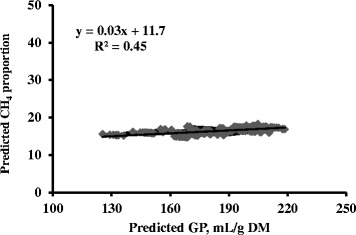
Relationship between gas production (GP) and methane (CH_4_) proportion (% CH_4_ on total GP) using the predicted values obtained from the mixed model analysis aimed to removing the study effect (i.e., the heterogeneity of variance among studies) and considering also the correction of raw data for the different accuracies^1^

Predicted values of in vitro GP and CH_4_ production showed a correlation of 0.90; the relationship obtained regressing in vitro predicted CH_4_ production against in vitro predicted GP produced a slope greater than 1 and a negative intercept (Fig. 1). The correlation between predicted values of in vitro GP and CH_4_ proportion was weaker (coefficient of determination, i.e., R^2^ = 0.45) (Fig. 2).

## Discussion

### General considerations

Over the last 10 years, in vitro GP technique has been largely adopted to evaluate fermentation properties of single feeds and diets for ruminants, as it is a fast and cost-effective procedure [10]. Up to now, several protocols of analysis are available, involving the use of different GP equipment, several incubation times, methods of rumen fluid collection, and different analytical procedures. For these reasons, values of GP and CH_4_ obtained from different in vitro experiments cannot be easily compared. Results of the present meta-analysis confirm that some experimental factors, which are individually discussed in the following sub-chapters, can influence measures of GP and CH_4_ obtained in vitro. However, it also emerged that methodological factors considered in the present meta-analysis allowed to explain only in part the variability of GP and CH_4_ values. For example, the two predictive equations obtained for in vitro measures of GP and CH_4_ showed a R^2^ of 0.48 and 0.34, respectively. It is likely that the inclusion of a higher number of factors in the model would have contributed to improve the accuracy of statistical predictions. Unfortunately, this was not possible as information about methodological aspects and setting of the experiment (i.e., laboratory procedures, animal and/or feed characteristics) were often missing or not exhaustive. For instance, in the present meta-analysis, more than half of studies comprised in the initial dataset (58 out of a total of 105 papers) were discarded because they did not report any information about one, or more, of methodological factors which have well-known effects on in vitro GP [10]. It is quite evident that a detailed description of the experimental procedures would also facilitate the comparison among results obtained in different researches.

### Pressure conditions in GP equipment

When in vitro equipment is used to measure GP and CH_4_, venting of gas is recommended to avoid overpressure conditions, which might disturb microbial activity [3] and cause a partial dissolution of CO_2_ in the fermentation fluid, thus underestimating GP measures [15]. From this meta-analysis it results that GP equipment operating at increasing pressure (i.e., without gas venting) provide, on average, lower measures of GP compared to those working at constant pressure. In contrast, values of CH_4_ proportion increased significantly when GP systems operating at increasing pressure were used. In this regard, [12] hypothesized that the increase of CO_2_ dissolved in the fermentation fluid, as result of overpressure conditions, would promote activity of methanogens. Additionally, when gas composition is analyzed, closed systems are often preferred, to avoid complexity of collecting vented gas into proper devices when open systems are used (i.e., gas-proof bags). With open systems, gas samples are collected from headspace of bottles at the end of incubation and analyzed for CH_4_ concentration [6, 7, 25]. These samples are considered to be most representative and to provide reliable measurements of CH_4_ because of the lower solubility in the fermentation fluid of CH_4_ compared to CO_2_ [6], hence measures are not affected by pressure changes in the bottles. According to this, the backward stepwise analysis did not highlight a significant effect of pressure on values of absolute CH_4_ production (mL/g DM). Differently, the CH_4_ proportion (% CH_4_ on total GP) was significantly influenced by pressure conditions in the GP system, resulting in greater values for equipment working at increasing pressure (closed systems). As recently observed [15], closed systems might underestimate in vitro GP, as a part of the CO_2_ is dissolved in the fermentation fluid, leading to a possible overestimation of the CH_4_ proportion on the total gas.

### Incubation time

The positive correlation between incubation time and values of in vitro GP was expected and it is related to the progressive degradation of feed sample incubated over longer incubation times. Likewise, the significant increase of CH_4_ production, in absolute terms, with the progress of in vitro fermentations is consistent with literature. For instance, [26] found that CH_4_ production (mL/g DM) of seven commercial diets for dairy cows increased by 106.5 % passing from 6 to 72 h of incubation. Similar results have been reported by [27] and by [28]. Such tendency is explained by the fact that CH_4_ formation is primarily related to fermentation of fibrous fraction that has a slower degradation rate compared to other dietary components. Results observed from the present meta-analysis might have been partially conditioned by the large predominance of roughages in the dataset considered. Nevertheless, it must be underlined that in vitro CH_4_ production is often evaluated at a single incubation time, thus less information are provided about the kinetics of CH_4_ formation in vitro. In this regard, only 5 of the 30 experiments considered in this meta-analysis measured CH_4_ production at different incubation times.

### Rumen fluid: collection time and donor species

Outcomes of this study showed that timing of rumen fluid collection had an impact on in vitro GP measures. More exactly, values of GP and CH_4_ production were greater when rumen fluid was collected after feeding the donor animals. This result might be explained by the presence of feed particles suspended in the rumen fluid, which can lead to an overestimation of the actual GP. This problem could be overcome through the incubation of blanks (bottles containing only the buffered rumen fluid), where the GP of rumen fluid can be determined and then used to adjust values of GP provided by experimental treatments [29]. However, [30] observed that microbial turnover begins more rapidly in blanks, thus they have a different GP rate compared to other treatments. On this basis, [31] discouraged the adjustment of GP data by using blank values.

This meta-analysis also shows that the time of rumen fluid collection is, to date, one of the least standardized procedures of the in vitro GP technique. This evidence is supported by the fact that some protocols of analysis used worldwide involve the collection of rumen fluid before feeding of donor animals [3, 32], whereas other authors suggest to feed animals before collection [2, 4]. In this regard, [1] indicated that rumen fluid used for in vitro tests should be collected before feeding of donor animals, as it has a less variable composition and, therefore, a more standardized effect on fermentations. However, the same authors [1] specified that the interval time occurring between the feeding of donors and the collection of rumen fluid should not exceed 16 h, to ensure a sufficient microbial activity in the inoculum to sustain in vitro GP.

The largest part of in vitro experiments considered in the present meta-analysis had been carried out using rumen fluid collected from bovine. Results indicate that values of GP and CH_4_ were greater when incubations were conducted using rumen fluid of bovine. Effects of donor species on in vitro GP are still uncertain, and a univocal ranking of various rumen fluids based on GP is not possible [10]. In recent years, several studies have been conducted to compare bovine and sheep rumen fluid, but results were contrasting [33–35]. However, GP values obtained using rumen fluid from different species might be reconciled by the appropriate use of blanks [1], although not all authors are in agreement with the possible correction for blanks [30, 31], as mentioned above. As a confirmation, in a ring test where rumen fluids collected from bovine or sheep were used [11], correction of GP data for the relative blank samples gave a notable reduction of variability between laboratories.

### Nitrogen in the buffer solution

The N in the buffer solution was found to be influential on in vitro GP and gas composition. More precisely, N-free buffers increased GP, CH_4_ production and proportion. To our knowledge, there is no evidence from literature that the buffer composition might influence CH_4_ measures obtained in vitro. The most experiments included in the dataset used a buffer solution containing N. In some cases, the composition of buffer used for in vitro tests is related to incubated feeds [10]. For instance, some buffers are rich in N and poor in energy sources, in order to evaluate energy contribution of feed samples to fermentations [1, 3]. On the opposite, other buffers are N-free, with the scope of evaluating the N contribution of high-protein feeds to in vitro fermentations [36]. It is likely that the buffer solution alone cannot modify in vitro GP and CH_4_ production in a significant way. More probably, some effects might appear when the mixture of buffer solution and feed sample is not balanced in terms of energy and N, thus microbial activity and growth might be impaired, with actual consequences on the various parameters of in vitro fermentation [10].

### Ratio between buffered rumen fluid and feed sample

Beuvink and Spoelstra [19] indicated that BRF/FS ratio must not exceed the proportion of 60 mL of buffered rumen fluid with 0.4 g OM of feed sample, which is 136 mL/g DM. According to these authors, such ratio can avoid the exhaustion of buffer and the drop of pH under the threshold of 6.2, which causes a nonlinear relation between feed sample size and GP [32]. On a total of 30 papers (339 observations), only 17 papers (169 observations) reported pH values of fermentation fluids measured at the end of incubation. Within these latter observations, 35 values were lower than the threshold of 6.2 (on average 5.98 ± 0.208; ranging from a minimum of 5.45 to a maximum of 6.19). However, in these experiments the drop of pH was not perforce related to a low BRF/FS ratio. This suggests that other factors (i.e., kind of buffer, kind of substrate, ratio between buffer solution and rumen fluid) could affect the pH trend during in vitro fermentation. Therefore, the actual effect of the BRF/FS ratio on GP and CH_4_ values is difficult to comment on. Furthermore, considering the dataset of this meta-analysis, it is evident that BRF/FS is one of the least standardized parameters for in vitro GP technique. Indeed, only 5 authors out of 30 followed the indications of [19], whereas 14 and 10 studies tested, respectively, lower (<130 mL/g DM) and higher (>140 mL/g DM) BRF/FS ratios. One study tested both lower and higher ratios. Results of the present meta-analysis show that the BRF/FS ratio was positively related to in vitro values of GP, CH_4_ production and proportion. In this regard, it could be hypothesized that, when the BRF/FS ratio increases, the fermentation fluid could be more capable of buffering the VFA produced from feed degradation, promoting the release of CO_2_ as indirect gas [9]. This process, in turn, would be expected to increase in vitro values of GP. Further, buffering action and maintenance of rumen pH would sustain the activity of methanogens, which are sensitive to acidification conditions [37], and thus the CH_4_ production might be increased.

### NDF content of feed samples

In line with our expectations, the NDF content of feeds incubated was one of the factors that affect in vitro measures of GP. More in detail, GP and NDF values were negatively correlated, as a high content of NDF in the feed is usually related to a reduced DM degradability [38] and, thus, to a low GP. For the same reason, the fibrous content of feeds was also negatively correlated with the absolute amount of CH_4_ produced in vitro (mL/g DM). It must be underlined that most of the data considered in the final database of the meta-analysis (273 out of the 339 observations) were represented by feed samples with a high NDF content (>300 g/kg DM). Such data distribution is likely to have increased the incidence of the effect attributable to the NDF content. In this regard, the lower absolute CH_4_ production (mL/g DM) of forages compared to concentrates, mainly due to the smaller extent of fermentation, was confirmed by several in vitro studies [6, 7, 15, 39].

## Conclusions

Results of this meta-analysis show that some methodological factors can notably influence in vitro measures of GP and CH_4_ production. It is evident that a full standardization of in vitro GP techniques is not feasible, as some of these factors (i.e., GP equipment, donor species of rumen fluid) are necessarily related to laboratory routine and facilities, and to the specific aim of the experiment. In any case, a greater harmonization of analytical procedures (i.e., a reduction in the number of available protocols) would be useful to facilitate comparison between results of different experiments. Further, exhaustive information about analytical procedures would be always included in the scientific papers. For instance, in the present meta-analysis, more than half of studies were excluded from the final dataset because they did not report some information about laboratory procedures and/or animal and feed characteristics that can influence fermentation patterns and, thus, measures of GP and CH_4_ obtained in vitro.

## Additional files

Additional file 1: Appendix 1 List of the publications excluded from the database. (DOC 43 kb) Additional file 2: Appendix 2 List of the publications included in the preliminary database. (DOC 40 kb)

## Footnotes

1 Adjustment for different accuracies of measurements in different studies was carried out by weighting raw data by the inverse of the squared standard error divided by the mean of all the squared standard errors (St-Pierre, 2001) [20].
