# Supplementary material for: Methodological factors affecting gas and methane production during in vitro rumen fermentation evaluated by meta-analysis approach
Source: J Anim Sci Biotechnol. 2016 Jun 14;7:35. doi: 10.1186/s40104-016-0094-8 (PMC4908760; doi:10.1186/s40104-016-0094-8)
Supplement: Additional file 1: — Appendix 1 List of the publications excluded from the database. (DOC 43 kb) [file 40104_2016_94_MOESM1_ESM.doc]

## Appendix 1. List of the publications excluded from the database

1. Abarghuei MJ, Rouzbehan Y, Salem AF. The influence of pomegranate-peel extracts on *in vitro* gas production kinetics of rumen inoculum of sheep. Turk. J. Vet. Anim. Sci. 2014;38:212-19.
2. Aemiro A, Watanabe S, Suzuki K, Hanada M, Umetsu K, Nishida T. Effects of Euglena (*Euglena gracilis*) supplemented to diet (forage: concentrate ratios of 60:40) on the basic ruminal fermentation and methane emissions in *in vitro* condition. Anim. Feed Sci. Technol. 2016;212:129-35.
3. Amelchanka SL, Kreuzer M, Leiber F. Utility of buckwheat (*Fagopyrum esculentum* Moench) as feed: Effects of forage and grain on *in vitro* ruminal fermentation and performance of dairy cows. Anim Feed Sci. Technol. 2010;155:111-21.
4. Anele UY, Sudeküm KH, Hummel J, Arigbede OM, Oni AO, Olanite JA, Böttger C, Ojo VO, Jolaosho AO. Chemical characterization, *in vitro* dry matter and ruminal crude protein degradability and microbial protein synthesis of some cowpea (*Vigna unguiculata L. Walp*) haulm varieties. Anim. Feed Sci. Technol. 2011;163:161-69.
5. Banik BK, Durmic Z, Erskine W, Nichols P, Ghamkhar K, Vercoe P. Variability of *in vitro* ruminal fermentation and methanogenic potential in the pasture legume biserrula (*Biserrula pelecinus L.*). Crop Pasture Sci. 2013;64(4):409-16.
6. Baraka TAM, Abdl-Rahman MA. *In vitro* evaluation of sheep rumen fermentation pattern after adding different levels of eugenol – fumaric acid combinations. Vet. World. 2012;5(2):110-17.
7. Becker PM, van Wikselaar PG. Effects of plant antioxidants and natural vicinal diketones on methane production, studied *in vitro* with rumen fluid and a polylactate as maintenance substrate. Anim. Feed Sci. Technol. 2011:170(3-4):201-08.
8. Blanco C, Bodas R, Prieto N, Moran L, Andres S, Lopez S, Giraldez FJ. Vegetable oil soapstocks reduce methane production and modify ruminal fermentation. Anim. Feed Sci. Technol. 2012;176(1-4):40-46.
9. Cao Y, Takahashi T, Horiguchi K, Yoshida N, Zhou D. *In vitro* ruminal dry matter digestibility and methane production of fermented total mixed ration containing whole-crop rice and rice bran. Grassl. Sci. 2012;58(3):133-39.
10. Castagnino PS, Messana JD, Fiorentini G, de Jesus RB, San Vito E, Carvalho IPC, Berchielli TT. Glycerol combined with oils did not limit biohydrogenation of unsaturated fatty acid but reduced methane production *in vitro*. Anim. Feed Sci. Technol. 2015;201:14-24.
11. Castro-Montoya J, De Campeneere S, Van Ranst G, Fievez V. Interactions between methane mitigation additives and basal substrates on *in vitro* methane and VFA production. Anim. Feed Sci. Technol. 2012;176(1-4):47-60.
12. Cobellis G, Petrozzi A, Forte C, Acuti G, Orrù M, Marcotullio MC, Aquino A, Nicolini A, Mazza V, Trabalza-Marinucci M. Evaluation of the effects of mitigation on methane and ammonia production by using *Origanum vulgare L.* and *Rosmarinus officinalis L.* essential oils on *in vitro* rumen fermentation systems. Sustainability 2015;7:12856-69.
13. Copani G, Ginane C, LeMorvan A, Niderkorn V. Patterns of *in vitro* rumen fermentation of silage mixtures including sainfoin and red clover as bioactive legumes. Anim. Feed Sci. Technol. 2015;208:220-24.
14. Durmic Z, Moate PJ, Eckard R, Revell DK, Williams R, Vercoe PE. *In vitro* screening of selected feed additives, plant essential oils and plant extracts for rumen methane mitigation. J. Sci. Food Agric. 2014;94(6):1191-96.
15. Elghandour MMMY, Kholif AE, Bastida AZ, Martinez DLP, Salem AZM. *In vitro* gas production of five rations of different maize silage and concentrate ratios influenced by increasing levels of chemically characterized extract of *Salix babylonica*. Turk. J. Vet. Anim. Sci. 2015;39:186-94.
16. Gemeda SB, Hassen A. Methane production of two roughage and total mixed ration as influenced by cellulose and xylanase enzyme addition. Sci. Agric. 2015;72(1):11-19.
17. Hart KJ, Yáñez-Ruiz DR, Duval SM, McEwan NR, Newbold CJ. Plant extracts to manipulate rumen fermentation. Anim. Feed Sci. Technol. 2008;147(1-3):8-35.
18. Hassim HA, Lourenco M, Goel G, Vlaeminck B, Goh YM, Fievez V. Effect of different inclusion levels of oil palm fronds on *in vitro* rumen fermentation pattern, fatty acid metabolism and apparent biohydrogenation of linoleic and linolenic acid. Anim. Feed Sci. Technol. 2010;162:155-58.
19. Hu WL, Liu JX, Ye JA, Wu YM, Guo YQ. Effect of tea saponin on rumen fermentation *in vitro*. Anim. Feed Sci. Technol. 2005;120(3-4):333-39.
20. Jayanegara A, Goel G, Makkar HPS, Becker K. Divergence between purified hydrolysable and condensed tannin effects on methane emission, rumen fermentation and microbial population *in vitro*. Anim. Feed Sci. Technol. 2015;209:60-68.
21. Kamalak A, Atalay AI, Ozkan CO, Tatliyer A, Kaya E. Effect of essential orange (*Citrus Sinensis l.*) oil on rumen microbial fermentation using *in vitro* gas production technique. J. Anim. Plant Sci. 2011;21(4):764-69.
22. Kim ET, Moon YH, Min KS, Kim CH, Kim SC, Ahn SK, Lee SS. Changes in microbial diversity, methanogenesis and fermentation characteristics in the rumen in response to medicinal plant extracts. Asian Australas. J. Anim. Sci. 2013;26(9):1289-94.
23. Lavrencic A, Levart A, Kosir IJ, Cerenak A. *In vitro* gas production kinetics and short-chain fatty acid production from rumen incubation of diets supplemented with hop cones (*Humulus lupulus* L.). Animal 2015;9(4):576-81.
24. Li X, Durmic Z, Liu S, McSweeney CS, Vercoe PE. *Eremophila glabra* reduces methane production and methanogen populations when fermented in a Rusitec. Anaerobe 2014;29:100-07.
25. Lin M, Schaefer DM, Zhao GQ, Meng QX. Effects of nitrate adaptation by rumen inocula donors and substrate fiber proportion on *in vitro* nitrate disappearance, methanogenesis, and rumen fermentation acid. Animal 2013;7(7):1099-1105.
26. Liu Y, Penuelas-Rivas CG, Buendia-Rodriguez G, Tan Z, Basurto-Gutierrez R, Wang M, Rivas-Guevara M. *In vitro* theoretic evaluation on ruminal fermentation performance by varying proportion supplementation of *Carthamus tinctorius* meal/*Brassica napus* seed with sorghum seed in ovine rations. J. Anim. Vet. Adv. 2015;14(2):43-53.
27. Lovett DK, Bortolozzo A, Conaghan P, O’Kiely P, O’Mara FP. *In vitro* total and methane gas production as influenced by rate of nitrogen application, season of harvest and perennial ryegrass cultivar. Grass Forage Sci. 2004;59(3):227-32.
28. Lovett DK, McGilloway D, Bortolozzo A, Hawkins M, Callan J, Flynn B, O’Mara FP. *In vitro* fermentation patterns and methane production as influenced by cultivar and season of harvest of *Lolium perenne L*. Grass Forage Sci. 2006;61(1):9-21.
29. Meale SJ, Chaves AV, Baah J, McAllister TA. Methane production of different forages in *in vitro* ruminal fermentation. Asian Australas. J. Anim. Sci. 2012;25(1):86-91.
30. Nanon A, Suksombat W, Yang WZ. Effects of essential oils supplementation on *in vitro* and *in situ* feed digestion in beef cattle. Anim. Feed Sci. Technol. 2014;196:50-59.
31. Naumann HD, Tedeschi LO, Muir JP, Lambert BD, Kothmann MM. Effect of molecular weight of condensed tannins from warm-season perennial legumes on ruminal methane production *in vitro*. Biochem. Syst. Ecol. 2013;50:154-62.
32. O’Brien M, Hashimoto T, Senda A, Nishida T, Takahashi J. The impact of *Lactobacillus plantarum* TUA1490L supernatant on *in vitro* rumen methanogenesis and fermentation. Anaerobe 2013;22:137-40.
33. Pang DG, Yang HJ, Cao BB, Wu TT, Wang JQ. The beneficial effect of *Enterococcus faecium* on the *in vitro* ruminal fermentation rate and extent of three typical total mixed rations in northern China. Livest. Sci. 2014;167:154-60.
34. Patra AK, Kamra DN, Agarwal N. Effect of spices on rumen fermentation, methanogenesis and protozoa counts in *in vitro* gas production test. Int. Congr. Ser. 2006;1293(7):176-79.
35. Pirondini M, Colombini S, Malagutti L, Rapetti L, Galassi G, Zanchi R, Crovetto GM. Effects of a selection of additives on *in vitro* ruminal methanogenesis and *in situ* and *in vivo* NDF digestibility. Anim. Sci. J. 2015;86:59-68.
36. Polyorach S, Wanapat M, Cherdthong A. Influence of Yeast Fermented Cassava Chip Protein (YEFECAP) and roughage to concentrate ratio on ruminal fermentation and microorganisms using *in vitro* gas production technique. Asian Australas. J. Anim. Sci. 2014;27(1):36-45.
37. Poulsen M, Jensen BB, Engberg RM. The effect of pectin, corn and wheat starch, inulin and pH on *in vitro* production of methane, short chain fatty acids and on the microbial community composition in rumen fluid. Anaerobe 2012;18:83-90.
38. Qiao JY, Tan ZL, Guan LL, Tang SX, Zhou CS, Han XF. Effects of hydrogen in headspace and bicarbonate in media on rumen fermentation, methane production and methanogenic population using *in vitro* gas production techniques. Anim. Feed Sci. Technol. 2015;206:19-28.
39. Rajkumar K, Bhar R, Kannan A, Jadhav RV, Singh B, Mal G. Effect of replacing oat fodder with fresh and chopped oak leaves on *in vitro* rumen fermentation, digestibility and metabolizable energy. Vet. World 2015;8(8):1021-26.
40. Rira M, Chentli A, Boufenera S, Bousseboua H. Effects of plants containing secondary metabolites on ruminal methanogenesis of sheep *in vitro*. Energy procedia 2015;74:15-24.
41. Rodrigues MAM, Lourenco AL, Cone JW, Nunes FM, Santos AS, Cordeiro JMM, Guedes CMV, Ferreira LMM. Evaluation of the nutritive value of muiumba (*Baikiaea plurijuga*) seeds: chemical composition, *in vitro* organic matter digestibility and *in vitro* gas production. Springerplus 2014;3:311-18.
42. Rojas Hernandez S, Olivares Perez J, Elghandour MMMY, Cipriano-Salazar M, Avila-Morales B, Camacho-Diaz LM, Salem AZM, Cerrillo Soto MA. Effect of polyethylene glycol on *in vitro* gas production of some non-leguminous forage trees in tropical region of the south of Mexico. Agroforest. Syst. 2015;89:735-42.
43. Rossi F, Vecchia P, Masoero F. Estimate of methane production from rumen fermentation. Nutr. Cycl. Agroecosys. 2001;60(1-3):89-92.
44. Salem AZM. Oral administration of leaf extracts to rumen liquid donor lambs modifies *in vitro* gas production of other tree leaves. Anim. Feed Sci. Technol. 2012;176:94-101.
45. Salem AZM, Kholif AE, Elghandour MMY, Hernandez SR, Dominguez-Vara IA, Mellado M. Effect of increasing levels of seven tree species extracts added to a high concentrate diet on *in vitro* rumen gas output. Anim. Sci. J. 2014;85(9):853-60.
46. Saminathan M, Chin Sieo C, Abdullah N, Vui Ling Wong CM, Wan Ho Y. Effects of condensed tannin fractions of different molecular weights from a *Leucaena* *leucocephala* hybrid on *in vitro* methane production and rumen fermentation. J. Sci. Food Agric. 2015;95(13):2742-49.
47. Serment A, Giger-Reverdin S, Schmidely P, Dhumez O, Broudiscou LP, Sauvant D. *In vitro* fermentation of total mixed diets differing in concentrate proportion: relative effects of inocula and substrates. J. Sci. Food Agric. 2016;96(1):160-68.
48. Soliva CR, Amelchanka SL, Duval SM, Kreuzer M. Ruminal methane inhibition potential of various pure compounds in comparison with garlic oil as determined with a rumen simulation technique (Rusitec). Br. J. Nutr. 2011;106(1):114-22.
49. Sun XZ, Hoskin SO, Muetzel S, Molano G, Clark H. Effects of forage chicory (*Cichorium intybus*) and perennial ryegrass (*Lolium perenne*) on methane emissions *in vitro* and from sheep. Anim. Feed Sci. Technol. 2011;166-167(6-7):391-97.
50. Tavendale MH, Meagher LP, Pacheco D, Walker N, Atwood GT, Sivakumaran S. Methane production from *in vitro* rumen incubations with *Lotus pedunculatus* and *Medicago sativa*, and effects of extractable condensed tannin fractions on methanogenesis. Anim. Feed Sci. Technol. 2005;123-124(1):403-19.
51. Theart JJF, Hassen A, van Niekerk WA, Gemeda BS. *In vitro* screening of Kalahari browse species for rumen methane mitigation. Sci. Agric. 2015;72(6):478-83.
52. Ungerfeld EM, Kohn RA, Wallace RJ, Newbold CJ. A meta-analysis of fumarate effects on methane production in ruminal batch cultures. J. Anim. Sci. 2007;85(10):2556-63.
53. Wallace RJ, Wood TA, Rowe A, Price J, Yáñez-Ruiz DR, Williams SP, Newbold CJ. Encapsulated fumaric acid as a means of decreasing ruminal methane emissions. Int. Congr. Ser. 2006;1293:148-51.
54. Wang Y, McAllister TA, Newbold CJ, Rode LM, Cheeke PR, Cheng KJ. Effects of *Yucca schidigera* extract on fermentation and degradation of steroidal saponins in the rumen simulation technique (RUSITEC). Anim. Feed Sci. Technol. 1998;74(2):143-53.
55. Williams CM, Eun JS, MacAdam JW, Young AJ, Fellner V, Min BR. Effects of forage legumes containing condensed tannins on methane and ammonia production in continuous cultures of mixed ruminal microorganisms. Anim. Feed Sci. Technol. 2011;166-167(6-7):364-72.
56. Wischer G, Boguhn J, Steingass H, Schollenberger M, Rodehutscord M. Effects of different tannin-rich extracts and rapeseed tannin monomers on methane formation and microbial protein synthesis *in vitro*. Animal 2013;7(11):1796-1805.
57. Wood TA, Wallace RJ, Rowe A, Price J, Yáñez-Ruiz DR, Murray P, Newbold CJ. Encapsulated fumaric acid as a feed ingredient to decrease ruminal methane emissions. Anim. Feed Sci. Technol. 2009;152(1-2):62-71.
58. Zhang H, Ji S, Chen Y, Yang B, Xu X, Hu C. Effect of α-ketoglutaric acid on *in vitro* gas production, ruminal fermentation, and bacterial diversity. Anim. Feed Sci. Technol. 2011;170:291-96.
