# Supplementary material for: Methodological factors affecting gas and methane production during in vitro rumen fermentation evaluated by meta-analysis approach
Source: J Anim Sci Biotechnol. 2016 Jun 14;7:35. doi: 10.1186/s40104-016-0094-8 (PMC4908760; doi:10.1186/s40104-016-0094-8)
Supplement: Additional file 2: — Appendix 2 List of the publications included in the preliminary database. (DOC 40 kb) [file 40104_2016_94_MOESM2_ESM.doc]

## Appendix 2 List of the publications included in the PRELIMINARY database

1. Amaro P, Maia MRG, Dewhurst RJ, Fonseca AJM, Cabrita ARJ. Effects of increasing level of stearidonic acid on methane production in a rumen *in vitro* system. Anim. Feed Sci. Technol. 2012;173(3-4):252-60.
2. Araujo RC, Pires AV, Mourau GB, Abdalla AL, Sallam SMA. Use of blanks to determine *in vitro* net gas and methane production when using rumen fermentation modifiers. Anim. Feed Sci. Technol. 2011;166-167(6-7):155-62.
3. Avila JS, Chaves AV, Hernandez-Calva M, Beauchemin KA, McGinn SM, Wang Y, Harstad OM, McAllister TA. Effects of replacing barley grain in feedlot diets with increasing levels of glycerol on *in vitro* fermentation and methane production. Anim. Feed Sci. Technol. 2011;166-167(6-7):265-68.
4. Bezabih M, Pellikaan WF, Tolera A, Khan NA, Hendriks WH. Chemical composition and *in vitro* total gas and methane production of forage species from the Mid Rift Valley grasslands of Ethiopia. Grass Forage Sci. 2014;69(4):635-43.
5. Bodas R, Lopez S, Fernandez M, Garcia-Gonzalez R, Rodriguez AB, Wallace RJ, Gonzalez JS. *In vitro* screening of the potential of numerous plant species as antimethanogenic feed additives for ruminants. Anim. Feed Sci. Technol. 2008;145(1-4):245-58.
6. Boguhn J, Zuber T, Rodehutscord M. Effect of donor animals and their diet on *in vitro* nutrient degradation and microbial protein synthesis using grass and corn silages. J. Anim. Physiol. Anim. Nutr. 2013;97(3):547-57.
7. Carrasco C, Medel P, Fuentetaja A, Carro MD. Effect of malate form (acid or disodium/calcium salt) supplementation on performance, ruminal parameters and blood metabolites of feedlot cattle. Anim. Feed Sci. Technol. 2012;176(1-4):140-49.
8. Cattani M, Tagliapietra F, Maccarana L, Hansen HH, Bailoni L, Schiavon S. Technical note: *In vitro* total gas and methane production measurements from closed or vented rumen batch culture systems. J. Dairy Sci. 2014;97(3):1736-41.
9. Elghandour MMY, Vázquez Chagoyán JC, Salem AZM, Kholif AE, Martínez Castaneda JS, Camacho LM, Cerrillo-Soto MA. Effects of *Saccharomyces cerevisiae* at direct addition or pre-incubation on *in vitro* gas production kinetics and degradability of four fibrous feeds. Ital. J. Anim. Sci. 2014;13(2):295-301.
10. Garcia-Gonzalez R, Giraldez FJ, Mantecon AR, Gonzalez JS, Lopez S. Effects of rhubarb (*Rheum spp.*) and franguala (*Frangula alnus*) on intake digestibility and ruminal fermentation of different diets and feedstuffs by sheep. Anim. Feed Sci. Technol. 2012;176(1-4):131-39.
11. Garcia-Gonzalez R, Lopez S, Fernandez M, Bodas R, Gonzalez JS. Screening the activity of plants and spices for decreasing ruminal methane production *in vitro*. Anim. Feed Sci. Technol. 2008a;147(1-3):36-52.
12. Garcia-Gonzalez R, Lopez S, Fernandez M, Gonzalez JS. Dose-response effects of *Rheum officinale root* and *Frangula alnus bark* on ruminal methane production *in vitro*. Anim. Feed Sci. Technol. 2008b;145(1-4):319-34.
13. Geerkens CH, Schweiggert RM, Steingass H, Boguhn J, Rodehutscord M, Carle R. Influence of apple and citrus pectins, processed mango peels, a phenolic mango peel extract, and gallic acid as potential feed supplements on *in vitro* total gas production and rumen methanogenesis. J. Agric. Food Chem. 2013;61(24):5727-37.
14. Getachew G, Robinson PH, DePeters EJ, Taylor SJ, Gisi DD, Higginbotham GE, Riordan TJ. Methane production from commercial dairy rations estimated using an *in vitro* gas technique. Anim. Feed Sci. Technol. 2005;123-124(1):391-402.
15. Guglielmelli A, Calabrò S, Primi R, Carone F, Cutrignelli MI, Tudisco R, Piccolo G, Ronchi B, Danieli PP. *In vitro* fermentation patterns and methane production of sainfoin (*Onobrychis viciifolia Scop.*) hay with different condensed tannin contents. Grass Forage Sci. 2011;66(4):488-500.
16. Hansen HH, Storm IMLD, Sell AM. Effect of biochar on *in vitro* rumen methane production. Acta Agr. Scand. A-an. 2013;62(4):305-09.
17. Hassanat F, Benchaar C. Assessment of the effect of condensed (acacia and quebracho) and hydrolysable (chestnut and valonea) tannins on rumen fermentation and methane production *in vitro*. J. Sci. Food Agric. 2013;93(2):332-39.
18. Hatew B, Cone JW, Pellikaan WF, Podesta SC, Bannink A, Hendriks WH, Dijkstra J. Relationship between *in vitro* and *in vivo* methane production measured simultaneously with different dietary starch sources and starch levels in dairy cattle. Anim. Feed Sci. Technol. 2015;202:20-31.
19. Holtshausen L, Chaves AV, Beauchemin KA, McGinn SM, McAllister TA, Odongo NE, Cheeke PR, Benchaar C. Feeding saponin-containing *Yucca schidigera* and *Qillaja saponaria* to decrease enteric methane production in dairy cows. J. Dairy. Sci. 2009;92(6):2809-21.
20. Kim DH, Mizinga KM, Kube JC, Friesen KG, McLeod KR, Harmon DL. Influence of monensin and lauric acid distillate or palm oil on *in vitro* fermentation kinetics and metabolites produced using forage and high concentrate substrate. Anim. Feed Sci. Technol. 2014;189:19-29.
21. Lee SY, Lee SM, Chi YB, Kam DK, Lee SC, Kim CH, Seo S. Glycerol as a feed supplement for ruminants: *In vitro* fermentation characteristic and methane production. Anim. Feed Sci. Technol. 2011;166-167(6-7):269-74.
22. Lila ZA, Mohammed N, Kanda S, Kamada T, Itabashi H. Effect of sarsaponin on ruminal fermentation with particular reference to methane production *in vitro*. J. Dairy. Sci. 2003;86(10):3330-36.
23. Lila ZA, Mohammed N, Tatsuoka N, Kanda S, Kurukawa Y, Itabashi H. Effect of cyclodextrin diallyl maleate on methane production, ruminal fermentation and microbes *in vitro* and *in vivo*. Anim. Sci. J. 2004;75(1):15-22.
24. Longo C, Bueno ICS, Nozella EF, Goddoy PB, Cabral Filho SLS, Abdalla AL. The influence of head-space and inoculum dilution on *in vitro* ruminal methane measurements. Int. Congr. Ser. 2006;1293(7):62-65.
25. Macheboeuf D, Morgavi DP, Papon Y, Mousset JL, Arturo-Schaan M. Dose-response effects of essential oils on *in vitro* fermentation activity of the rumen microbial population. Anim. Feed Sci. Technol. 2008;145(1-4):335-50.
26. Martínez ME, Ranilla MJ, Tejido ML, Saro C, Carro MD. The effect of the diet fed to donor sheep on *in vitro* methane production and ruminal fermentation of diets of variable composition. Anim. Feed Sci. Technol. 2010;158(3-4):126-35.
27. Narvaez N, Wang Y, McAllister T. Effects of extracts of *Humulus lupulus* (hops) and *Yucca schidigera* applied alone or in combination with monensin on rumen fermentation and microbial populations *in vitro*. J. Sci. Food Agric. 2013;93(10):2517-22.
28. Navarro-Villa A, O’Brien M, López S, Boland TM, O’Kiely P. *In vitro* rumen methane output of red clover and perennial ryegrass assayed using the gas production technique (GPT). Anim. Feed Sci. Technol. 2011a;168(3-4):152-64.
29. Navarro-Villa A, O’Brien M, López S, Boland TM, O’Kiely P. Modifications of a gas production technique for assessing *in vitro* rumen methane production from feedstuffs. Anim. Feed Sci. Technol. 2011b;166-167(6-7):163-74.
30. O’Brien MO, Navarro-Villa A, Purcell PJ, Boland TM, O’Kiely PO. Reducing *in vitro* rumen methanogenesis for two contrasting diets using a series of inclusion rates of eleven additives. Anim. Prod. Sci. 2014;54(2):141-57.
31. Pal K, Patra AK, Sahoo A, Kumawat PK. Evaluation of several tropical tree leaves for methane production potential, degradability and rumen fermentation *in vitro*. Livest. Sci. 2015;180:98-105.
32. Pal K, Patra AK, Sahoo A, Mandal GP. Effect of nitrate and fumarate in *Prosopis cineraria* and *Ailanthus excelsa* leaves-based diets on methane production and rumen fermentation. Small Rum. Res. 2014;121(2-3):168-74.
33. Patra AK, Yu Z. Effective reduction of enteric methane production by a combination of nitrate and saponin without adverse effect on feed degradability, fermentation, or bacterial and archaeal communities of the rumen. Bioresour. Technol. 2013a;148:352-60.
34. Patra AK, Yu Z. Effects of gas composition in headspace and bicarbonate concentrations in media on gas and methane production, degradability, and rumen fermentation using *in vitro* gas production techniques. J. Dairy Sci. 2013b;96(7):4592-4600.
35. Pellikaan WF, Hendriks WH, Uwimana G, Bongers LJGM, Becker PM, Cone JW. A novel method to determine simultaneously methane production during *in vitro* gas production using fully automated equipment. Anim. Feed Sci. Technol. 2011;168(3-4):196-205.
36. Pirondini M, Malagutti L, Colombini S, Amodeo P, Crovetto GM. Methane yield from dry and lactating cows diets in the Po Plain (Italy) using an *in vitro* gas production technique. Ital. J. Anim. Sci. 2012;11(3):330-35.
37. Purcell PJ, O’Brien M, Boland TM, O’Donovan M, O’Kiely P. Impacts of herbage mass and sward allowance of perennial ryegrass sampled throughout the growing season on *in vitro* rumen methane production. Anim. Feed Sci. Technol. 2011a;166-167(6-7):405-11.
38. Purcell PJ, O’Brien M, Boland TM, O’Kiely P. *In vitro* rumen methane output of perennial ryegrass samples prepared by freeze drying or thermal drying (40°C). Anim. Feed Sci. Technol. 2011b;166-167(6-7):175-82.
39. Ramin M, Huhtanen P. Development of an *in vitro* method for determination of methane production kinetics using a fully automated *in vitro* gas system - A modelling approach. Anim. Feed Sci. Technol. 2012;174(3-4):190-200.
40. Ramin M, Krizsan SJ, Jančík F, Huhtanen P. Short communication: measurements of methane emissions from feed samples in filter bags or dispersed in the medium in an *in vitro* gas production system. J. Dairy Sci. 2013;96(7):4643-46.
41. Ramin M, Lerose D, Tagliapietra F, Huhtanen P. Comparison of rumen fluid inoculum vs. faecal inoculum on predicted methane production using a fully automated *in vitro* gas production system. Livest. Sci. 2015;181:65-71
42. Sallam SMA, Bueno ICS, Nasser MEA, Abdalla AL. Effect of eucalyptus (*Eucalyptus citriodora*) fresh or residue leaves on methane emission *in vitro*. Ital. J. Anim. Sci. 2010;9(3):299-303.
43. Soliva CR, Zeleke AB, Clement C, Hess HD, Fievez V, Kreuzer M. *In vitro* screening of various tropical foliages, seeds, fruits and medicinal plants for low methane and high ammonia generating potentials in the rumen. Anim. Feed Sci. Technol. 2008(1-3);147:53-71.
44. Theodoridou K, Aufrère J, Niderkorn V, Andueza D, Le Morvan A, Picard F, Baumont R. *In vitro* study of the effects of condensed tannins in sainfoin on the digestive process in the rumen at two vegetation cycles. Anim. Feed Sci. Technol. 2011;170:147-59.
45. Tuyen DV, Phuong HN, Cone JW, Baars JJP, Sonnenberg ASM, Hendriks WH. Effect of fungal treatments of fibrous agricultural by-products on chemical composition and *in vitro* rumen fermentation and methane production. Bioresour. Technol. 2013;129:256-63.
46. Xu M, Rinker M, McLeod KR, Harmon DL. *Yucca schidigera* extract decreases *in vitro* methane production in a variety of forages and diets. Anim. Feed Sci. Technol. 2010;159(1-2):18-26.
47. Zhang DF, Yang HJ. *In vitro* ruminal methanogenesis of a hay-rich substrate in response to different combination supplements of nitrocompounds; pyromellitic diimide and 2-bromoethanesulphonate. Anim. Feed Sci. Technol. 2011;163:20-32.
